# Utility of Transcranial Magnetic Simulation in Studying Upper Motor Neuron Dysfunction in Amyotrophic Lateral Sclerosis

**DOI:** 10.3390/brainsci11070906

**Published:** 2021-07-09

**Authors:** Nimeshan Geevasinga, Mehdi Van den Bos, Parvathi Menon, Steve Vucic

**Affiliations:** Western Clinical School, University of Sydney, Sydney, NSW 2006, Australia; mehdi.vandenbos@health.nsw.gov.au (M.V.d.B.); parmenon2010@gmail.com (P.M.); s.vucic@neura.edu.au (S.V.)

**Keywords:** Amyotrophic lateral sclerosis, transcranial magnetic stimulation, review

## Abstract

Amyotrophic lateral sclerosis (ALS) is characterised by progressive dysfunction of the upper and lower motor neurons. The disease can evolve over time from focal limb or bulbar onset to involvement of other regions. There is some clinical heterogeneity in ALS with various phenotypes of the disease described, from primary lateral sclerosis, progressive muscular atrophy and flail arm/leg phenotypes. Whilst the majority of ALS patients are sporadic in nature, recent advances have highlighted genetic forms of the disease. Given the close relationship between ALS and frontotemporal dementia, the importance of cortical dysfunction has gained prominence. Transcranial magnetic stimulation (TMS) is a noninvasive neurophysiological tool to explore the function of the motor cortex and thereby cortical excitability. In this review, we highlight the utility of TMS and explore cortical excitability in ALS diagnosis, pathogenesis and insights gained from genetic and variant forms of the disease.

## 1. Introduction

Amyotrophic lateral sclerosis is a progressive neurodegenerative disorder first described by Charcot [1,2]. The incidence of ALS has been estimated at two per hundred thousand [3], with a median survival of 3–5 years [4,5]. The disease can start focally and spread insidiously to other regions over time with respiratory muscle weakness heralding the eventual demise of patients [6]. The majority of ALS patients (65–75%) present with asymmetrical weakness, leading to wasting of the limb muscles, typically spreading along the neuroaxis and affecting contiguous motor neurons [7,8,9]. In 20% of ALS patients the disease can involve the bulbar muscles, presenting with progressive dysphagia and dysarthria [10]. Neuronal degeneration in ALS encompasses both the central and peripheral nervous systems, leading to the pathognomonic clinical features of ALS with a combination of upper (UMN) and lower motor (LMN) neuron clinical examination findings. [11].

Cortical hyperexcitability appears to be an early feature of ALS, highlighting the importance of the UMN dysfunction in the disease process [2,12]. Transcranial magnetic stimulation is a noninvasive tool that allows studying UMN dysfunction and thereby cortical hyperexcitability utilising novel neurophysiological parameters [13]. Cortical hyperexcitability is a unique and specific feature of ALS, differentiating the pathophysiological processes in ALS from other mimic disorders such as Kennedy’s disease, oculopharyngeal muscular dystrophy, multifocal motor neuropathy and spinal muscular atrophy [12,14].

Whilst the underlying pathogenic process in ALS is still unclear, three different hypotheses have been proposed. After the initial description by Charcot [1], Eisen and colleagues proposed the “dying forward” hypothesis, where hyperexcitability of descending corticomotoneuronal tracts resulted in motor neuron degeneration via an anterograde transsynaptic glutamate excitotoxic process [15]. Clinical observations have supported this dying forward mechanism including: (i) Sparing of muscles with a sparse corticomotoneuronal supply (extraocular and sphincter muscles) and clinical appearances of the split-hand pattern [16]; (ii) ALS phenotypes with pure LMN findings were infrequent [17]; and (iii) lack of ALS in the animal world, postulated due to developmental differences in corticomotoneuronal projections onto the spinal motor neurons across species [18]. In contrast, the primacy of LMNs in ALS pathogenesis have been suggested in the dying-back hypothesis [19,20], albeit only supported by animal models. Additionally, the independent degeneration hypothesis was also advanced which suggested that UMN and LMN degeneration occurs independently [21].

Supporting the notion for the importance of UMN dysfunction in ALS, recent clinical, neurophysiological, radiological and genetic advances, have suggested the importance of cortical hyperexcitability in ALS pathogenesis. Given the diagnostic challenges seen in ALS, cortical hyperexcitability may be an early diagnostic biomarker in ALS, allowing earlier diagnosis of ALS patients [22]. In this review, we highlight the utility of TMS in identifying cortical hyperexcitability in ALS, furthering the understanding of the pathophysiological processes driving ALS, aiding in the diagnosis of this condition and the utility cortical hyperexcitability as a diagnostic biomarker in this progressive and invariably fatal disease.

### 1.1. Cortical Hyperexcitability

To explore and assess the function of the motor pathways in ALS and thereby gaining better understanding of ALS pathophysiology, TMS can be utilised to assess cortical excitability. The utility of single, paired and triple-pulse transcranial magnetic stimulation (TMS) techniques have significantly enhanced the understanding of cortical hyperexcitability in ALS pathophysiology, thereby resulting in novel diagnostic approaches [23]. TMS is a neurophysiological technique which is performed non-invasively by stimulating the human motor cortex; this technique was first described by Barker and colleagues [24]. TMS activates the motor cortex at a depth of approximately 1.5 to 2.1 cm [25], resulting in the generation of direct (D) and/or indirect (I) waves [26,27], reflecting the function of cortical output cells (Betz cells) and intracortical neuronal networks within the primary motor cortex (M1). In a clinical setting, to assess cortical excitability and evaluate the integrity of corticospinal pathways, the following TMS parameters are most useful: (i) short interval intracortical inhibition; (ii) motor threshold; (iii) motor evoked potential (MEP) amplitude; (iv) cortical silent period (CSP) duration and (v) short interval intracortical facilitation (SICF), (Table 1).

Short interval intracortical inhibition (SICI) is the most robust TMS parameter of cortical excitability [22], and is generated by the paired-pulse TMS paradigm where a subthreshold conditioning stimulus precedes a suprathreshold test stimulus at a preset interstimulus interval (ISI) [13,28]. Using this paradigm, when TMS pulses are delivered between an ISI of 1–5 ms, the test response is inhibited, thereby generating SICI. As the ISI is increased to intervals of 7 and 30 ms, a facilitation of the test responses is noted resulting in intracortical facilitation (ICF) [13]. In the original constant stimulus technique, the condition and test stimulus intensity were kept constant, but this technique was affected by MEP amplitude variability [29,30]. The threshold tracking TMS technique overcame this limitation by maintaining a constant target MEP response (0.2 mV) and varying the suprathreshold stimulus test intensity [13,31]. Physiological studies exploring the generation of SICI have shown it is mediated by inhibitory GABAergic intracortical circuits acting via GABA_A_ intracortical circuits, located within the primary motor cortex [26,27]. In addition, a weak effect of glutaminergic neurotransmission has also been reported [32].

A reduction, or absence of SICI in sporadic ALS patients has been reported as indicative of cortical hyperexcitability [33,34,35,36,37]. The reduction in SICI is an early feature of ALS; it precedes the onset of LMN dysfunction and correlates with neurophysiological biomarkers of peripheral neurodegeneration [22,35,38]. Abnormalities of SICI have also been associated with patterns of disease spread [39,40] and development of clinical features seen in ALS such as the split-hand phenomenon [16]. Reduction of SICI was also evident in atypical sporadic ALS phenotypes, including patients with pure LMN syndromes, thereby indicating subclinical UMN dysfunction [41].

The CSP duration is a distinct neurophysiological biomarker of cortical excitability. It is mediated by long-lasting inhibitory post-synaptic potentials generated via GABA_B_ receptors [42]. The CSP duration can also be modulated by the density of the corticomotoneuronal projections onto motor neurons, motor attention, the extent of voluntary motor drive and neurotransmitters such as dopamine [32,42]. Reduction of CSP duration has been noted in sporadic and familial ALS cohorts, being most prominent in early disease stages [34,35,36,37,43,44]. Abnormalities of ipsilateral CSP have also been reported as an early feature in ALS, signifying loss of transcallosal function [44,45]. Disinhibition at a cortical level, due to degeneration or dysfunction of cortical inhibitory interneurons, appears to underlie the reduction of CSP duration in ALS [45].

Marked changes in motor thresholds have been also described in ALS [23]. The resting motor threshold reflects the density of corticomotoneuronal projections onto the spinal motor neurons as well as cortical excitability, influenced by glutamatergic neurotransmission and Na^+^ channel conductance [23,25,32]. When motor thresholds have been studied longitudinally in TMS studies, ALS patients had reduced motor thresholds in the early stages of the disease, progressing to an increase in motor threshold and eventual cortical inexcitability [34,35,37,46,47,48]. This early reduction in motor thresholds, likely reflects cortical hyperexcitability, and appears to be most prominent in patients with profuse fasciculations, exaggerated deep-tendon reflexes and preserved muscle bulk [49], thereby supporting the notion that fasciculations are derived from cortical dysfunction in the early stages of ALS [50] and that cortical hyperexcitability may underlie the development of neurodegeneration in ALS. Similarly, increased MEP amplitudes have been noted in ALS [23]. Similar to motor thresholds, the MEP amplitude reflects the density of corticomotoneuronal projections onto motor neurons [51], and it appears to be influenced by neurotransmitters such as glutamate [23,51]. Increases in MEP amplitude have been reported as an early feature in ALS [35,37,41], correlating with surrogate neurophysiological biomarkers of axonal degeneration.

Another novel neurophysiological parameter, SICF, is also generated by the paired-pulse TMS technique. This neurophysiological parameter is obtained when a conditioning stimulus is set to peri- and suprathreshold levels followed by a test stimulus set at threshold intensity [52,53]. The threshold tracking TMS technique has been utilised to generate SICF, revealing two distinct peaks at ISI of 1.5 and 3 ms [54]. Whilst the exact physiological processes underlying SICF development still remain undetermined, a cortical origin has been proposed with SICF likely reflecting activity of facilitatory cortical circuits [54,55]. Increases in SICF were reported in sporadic ALS patients, noticed in combination with a reduction in SICI [56]. A novel index of excitation, a neurophysiological biomarker of cortical excitability expressing SICF as a function of SICI, was recently described, revealing an increased index of excitation in ALS patients, suggesting overactivity of facilitatory circuits contributing to cortical hyperexcitability [57].

Other more complex TMS stimulation paradigms include the triple stimulation technique, which has also shown utility in detecting sub-clinical UMN dysfunction in the early stages of ALS [58,59].

Some have argued that cortical hyperexcitability is a compensatory mechanism secondary to motor neuron degeneration [34]. However, given that cortical hyperexcitability was not evident in ALS mimicking disorders, despite a comparable degree of LMN burden [12,22,36,43], this would argue against a compensatory mechanism. In addition, the partial normalization of cortical hyperexcitability with riluzole [60], an anti-glutaminergic agent which has modest therapeutic benefits in ALS, provides further support for the pathogenic role of cortical hyperexcitability in ALS. The presence of cortical hyperexcitability as measured as reductions in SICI has utility as a prognostic marker, predicting a worse prognosis in an ALS cohort [61].

### 1.2. The Split-Hand Pattern

A role for corticomotoneuronal dysfunction in ALS pathogenesis is supported by the clinical observation that the split-hand pattern of muscle wasting is a specific feature in ALS [16]. The split-hand phenomenon refers to preferential wasting of the intrinsic hand muscles the abductor pollicis brevis (APB) and first dorsal interosseous (FDI) muscles, when compared to the hypothenar muscles [62]. This dissociated pattern of intrinsic hand muscle atrophy is specific for ALS and is not noted in ALS mimic disorders [63,64]. Transcranial magnetic studies have shown that cortical hyperexcitability appears to underlie the development of the split-hand phenomenon [16], suggesting a greater cortical representation of the thenar muscles, utilised for fine precision tasks, thereby reaffirming the importance of UMN dysfunction in ALS [18,65]. Additionally, cortical hyperexcitability was reported to be a specific feature of the split-hand plus phenomenon, where preferential weakness of the thenar muscle is observed when compared to flexor pollicis brevis [66,67]. More recently, a split-leg (preferential weakness of posterior calf muscles) and a split-elbow (preferential weakness of the biceps brachii compared to the triceps muscle) phenomenon were reported as specific clinical features of ALS and also attributed to cortical dysfunction [68,69].

### 1.3. Insights Gathered from Studying Cortical Excitability in Familial ALS

The recent advances in the genetic understanding of ALS have provided further insights into the role of the corticomotor neurons. In particular, the identification of an increased hexanucleotide repeat expansion (GGGGCC) in the c9orf72 gene on chromosome 9p21.1 [70,71] which accounts for over 40% of familial and approximately 8% of apparently “sporadic” ALS cases [70,71,72] has radically altered the understanding of ALS pathogenesis. The c9orf gene expansion is also the most common cause of familial frontotemporal dementia, thereby placing ALS in the continuum of an underlying cortical neurodegenerative disorder [70]. At one end of the spectrum of this gene mutation lies frontotemporal dementia, on the other end ALS, with some patients presenting with a mixed picture of cognitive and motor deficits, FTD–ALS. The overwhelming majority of ALS cases and approximately 50% of FTD cases are characterized by inclusions consisting of the RNA-binding protein TDP-43 (TAR DNA-binding protein 43) in neurons and glia [73], suggesting a shared pathogenic process. The role of cortical dysfunction in ALS is further evident with studies showing that up to 30–50% of ALS patients present with cognitive dysfunction, suggesting extra motor involvement [74,75].

Specifically SICI reduction appears to be an early and prominent feature in familial ALS, including patients expressing mutations in the superoxide dismutase-1 [37], fused in sarcoma (FUS) [76] and c9orf72 expansion [77], and correlating with peripheral neurodegeneration [78]. The presence of cortical hyperexcitability was noted in a cohort of c9orf72 FALS patients, but not in asymptomatic c9orf72 expansion carriers [77]. These findings confirm that cortical hyperexcitability is a feature of familial forms of ALS and suggests that it develops prior to clinical onset of FALS.

As such, irrespective of the underlying genetic mutation in familial ALS patients, cortical hyperexcitability appears to represent a uniform pathophysiological process in ALS. Of relevance, recent studies looking at complex mathematical modeling of ALS pathophysiology have inferred a six-step process in ALS [79], with a prolonged prodromal period, potentially extending to the perinatal period [80], where patients may develop an increased risk of developing ALS in utero. This multistep model of disease progression in ALS was also recently suggested in another study looking at Australia, Japanese and Korean patients [81]. Cortical hyperexcitability probably represent an important step in ALS pathogenesis, developing just prior to or at onset of neuronal degeneration. This notion is supported by findings of significant correlations between neurophysiological features of cortical hyperexcitability and motor amplitude and clinically measured muscle strength scores. Recent animal studies further support this notion, identifying neuronal hyperexcitability at a pre-clinical stage, upstream of the spinal motor neurons [82,83].

### 1.4. The Role of TMS in Aiding Diagnosis

The current diagnosis of ALS relies on the clinical and neurophysiological identification of a combination of UMN and LMN signs in multiple body regions, along with evidence of ongoing disease progression noted over time. Importantly, in confirming ALS, any mimic disorders need to be excluded prior to establishing a definitive diagnosis of ALS [6,84]. The clinically based ALS diagnostic criteria (El-escorial and the Awaji criteria) can be insensitive, particularly in the early stages of the disease or in the setting of atypical ALS phenotypes. This can then unfortunately result in significant diagnostic delays [85]. Consequently, delayed diagnosis may slow the implementation of adequate management strategies, including commencement of therapies such as riluzole, and recruitment into therapeutic trials, perhaps beyond the therapeutic window period [86]. The recently reported Gold Coast criteria aim to simplify diagnostic criteria by requiring UMN and LMN dysfunction in at least one body region or two regions with LMN findings [87]. The clinical identification of UMN signs in ALS may be limited by complex physiological factors, including marked muscle wasting along with dysfunction of descending motor pathways and local spinal circuits that usually facilitate UMN signs [88]. Separately, adaptive changes within the neuromuscular system may further complicate assessment.

Consequently, direct assessment of UMN function by TMS techniques could overcome the limitations of the disease process, providing an objective diagnostic biomarker of UMN dysfunction. Studies have shown that utilising TMS may allow identifying a greater proportion of patients with ALS at an earlier stage [22,89]. The most robust marker in the aid of diagnosing ALS was a reduced SICI or an inexcitable motor cortex [22,89]. Other changes documented included reductions in resting motor threshold and CSP duration, along with an increases in MEP amplitude and ICF [89].

### 1.5. Utilising TMS to Differentiate between Clinical Disorders

Whilst the most common phenotype of ALS includes involvement of both the UMN and LMNs, there are other forms of the disease that can present with atypical phenotypes. One particular variant includes predominant involvement of the UMNs, termed primary lateral sclerosis (PLS), which is characterized by a slowly progressive UMN syndrome with absence of LMN features for up to four years [90,91,92,93]. Utilising TMS, a greater frequency of motor cortex inexcitability was noted in the PLS phenotype than in ALS patients [14]. This finding is probably related to a greater degree of neurodegeneration within the motor cortex and the corticospinal tracts, as the resting motor threshold reflects the density of corticomotoneuronal projections onto spinal motor neurons as well as excitability of large motor cortical neurons (Betz cells) [23]. Furthermore, TMS is useful in differentiating ALS from a mimic disorder such as hereditary spastic paraparesis, which does not have any evidence of cortical dysfunction as measured by TMS [14].

Another atypical variant of ALS is the flail leg (FL) syndrome also referred to as leg amyotrophic diplegia; this phenotype was first described in the early 20th century [94]. Clinically, the FL syndrome is characterised by a predominant LMN phenotype with absent or subtle upper motor neuron features. The disease remains limited to the lower limbs for a prolonged period [94,95,96]. The median survival for the FL phenotype is more favourable that the typical ALS phenotype, estimated to be 90 months [94,95]. There appears to be a heterogeneity of the FL phenotype, and some studies have reported a greater degree of UMN dysfunction and shorter survival, approximating the disease course of the more classical ALS [97]. Cortical hyperexcitability measured as a significant reduction of SICI, was only evident in the FL patients exhibiting UMN signs, with the degree of cortical hyperexcitability similar to other ALS phenotypes [98]. In contrast, in FL patients with absent UMN signs, cortical excitability was preserved, despite a prolonged central motor conduction time in the entire cohort. These findings suggest heterogeneity in the clinical phenotype and underlying pathophysiological processes of FL patients. Similarly, in patients with the flail arm variant, cortical excitability was evident similar to patients with classical ALS with reductions in SICI and resting motor threshold documented [41].

## 2. Conclusions

Cortical hyperexcitability is an important pathophysiological and diagnostic biomarker in sporadic and familial ALS. Cortical hyperexcitability is heralded by development of cortical disinhibition and increases in activity of cortical facilitatory circuits with evolution of the disease course. It precedes the development of LMN dysfunction and correlates with neuronal degeneration. Future studies looking at transcranial magnetic stimulation-electroencephalography, molecular/proteomics, genetic studies evaluating familial forms of ALS and advanced neuroimaging techniques may help provide further insights into the pathophysiological mechanisms underlying ALS. A better understanding of the underlying pathophysiological processes could lead to identification of novel pathogenic biomarkers that serve as therapeutic targets and be utilised as biomarkers in measuring disease activity or disease progression in future ALS therapeutic trials.

## Figures and Tables

**Table 1:** Physiological mechanisms underlying transcranial magnetic stimulation (TMS) neurophysiological parameters in ALS patients.

TMS Neurophysiological Parameters	Suspected Physiological Mechanisms
Motor threshold	Density of corticomotoneuronal projections on spinal motor neurons as well as cortical excitability being influenced by glutamatergic neurotransmission and Na^+^ channel conductance.
Motor evoked potentials (MEP)	Summation of complex corticospinal volleys consisting of D and/or I waves onto the spinal motor neuron.
Central motor conduction time (CMCT)	Prolongation of the CMCT probably reflects degeneration of the fastest conducting corticomotoneuronal axons in ALS patients, resulting in increased desynchronization of corticomotoneuronal volleys due to axonal loss.
Cortical silent period (CSP)	Probably multifactorial in nature, with the early segment mediated by spinal processes, whilst the later segment being mediated by long-lasting inhibitory post-synaptic potentials generated via GABAergic B receptors.
Short interval intracortical inhibition (SICI)	Mediated by inhibitory GABAergic intracortical circuits located within the primary motor cortex, acting via GABA_A_ receptors.
Intracortical facilitation (ICF)	Generated at the motor cortex, associated with increases in the I-wave amplitude, being reduced by GABA_A_ receptor agonists.
Short interval intracortical facilitation (SICF)	A cortical origin has been proposed reflecting activity of facilitatory cortical circuits, potentially from axonal–axonal reinforcement of interneuronal activations resulting in synergistic increases in corticospinal output.
